# COVID-19 and Pulmonary Embolism: Diagnostic Imaging Trends

**DOI:** 10.2967/jnumed.120.248518

**Published:** 2020-08

**Authors:** Sedighe Karimzadeh, Akshay Raut, Nguyen Tien Huy

**Affiliations:** * School of Tropical Medicine and Global Health, 12 Nagasaki 852-8102, Japan E-mail: tienhuy@nagasaki-u.ac.jp

**TO THE EDITOR:** A recently published editorial by Zuckier et al. (1), titled “Diagnostic Evaluation of Pulmonary Embolism During the COVID-19 Pandemic,” suggested reverting to a nonventilation approach for the evaluation of pulmonary embolism (PE) to minimize potential exposure to aerosolized secretions in the nuclear medicine suite.

Although the authors have provided a novel approach to mitigate the risk of aerosolized transmission from coronavirus disease 2019 (COVID-19) patients, the diagnostic efficacy of the algorithm needs to improve. First, the authors have mentioned reducing the number of ventilation scans by rigorous assessment of pretest probability using well-known diagnostic scoring systems such as the Wells criteria, the PE rule-out criteria, and the Geneva scoring system. Although these scoring systems are commonly used to predict PE in the outpatient population, they might not be an appropriate and valid tool to predict the risk of PE in COVID-19 patients who are critically ill or admitted in the intensive care unit, which is attributed to the higher mortality rate (2–4). In this context, if not contraindicated, cardiothoracic pulmonary angiography is recommended as the instant diagnostic tool regardless of chest radiography findings (5). Consequently, it is crucial to consider patients’ hemodynamic instability before approaching the mentioned algorithm.

In the setting of infection with severe acute respiratory syndrome coronavirus 2 and a contraindication to cardiothoracic pulmonary angiography, respiratory distress in COVID-19 patients may preclude an optimal ventilation–perfusion scan procedure. In such cases, we recommend reverting to perfusion-only scintigraphy or bedside critical-care ultrasound as a real-time point of care examination (if the availability of scintigraphy is limited), along with modified scoring system, clinical judgment, and D-dimer assay. Although a positive value for D-dimer does not significantly predict the risk of PE, a negative D-dimer test (<500 ng/mL) has a high negative predictive value when there is a low or intermediate pretest likelihood (6–8). A negative result for D-dimer test can reduce the number of imaging modalities, leading to minimized aerosolized secretions in ventilation–perfusion scans.

Marked increase in coagulation parameters including D-dimer level, fibrin degradation products, and fibrinogen are reportedly associated with a higher mortality rate in COVID-19 patients (3,9). With an increasing hypercoagulability state in COVID-19 patients in the absence of major predisposing factors (in scoring assessments), such as previous proven deep-vein thrombosis or PE, recent major surgery or trauma, pregnancy, or cancer (which result in a low risk-probability for PE as per the scoring systems), these patients can still probably have PE. According to the criteria of the Prospective Investigative Study of Acute PE Diagnosis, perfusion scintigraphy has inferior diagnostic value than combined ventilation–perfusion scintigraphy in patients with a low pretest probability for PE (10), which occurs and is expected more frequently in COVID-19 patients as explained earlier.

Ultimately, a negative perfusion-only scintigraphy result cannot reliably exclude PE in all COVID-19 patients. Other imaging techniques, clinical judgment, and laboratory evaluations are reconsidered to efficiently diagnose PE in these patients.
